# Assessing multidimensional care coverage for pre‐eclampsia in the era of universal health coverage: A pre–post evaluation of the Salud Mesoamérica Initiative

**DOI:** 10.1002/ijgo.13131

**Published:** 2020-03-25

**Authors:** Aruna M. Kamath, Casey K. Johanns, Maximillian G. Thom, Rebecca M. Cogen, Diego Rios‐Zertuche, Ali H. Mokdad, Bernardo Hernández

**Affiliations:** ^1^ Institute for Health Metrics and Evaluation University of Washington Seattle WA USA; ^2^ Department of Anesthesiology University of Washington Seattle WA USA; ^3^ Inter‐American Development Bank Washington DC USA

**Keywords:** Belize, Central America, Eclampsia, Honduras, Maternal health, Nicaragua, Pre‐eclampsia, Quality of health care

## Abstract

**Objective:**

To compare a multidimensional care package for pre‐eclampsia/eclampsia in Central American health facilities, before and after implementation of the Salud Mesoamérica Initiative.

**Methods:**

An evaluation study was conducted at 67 basic‐ and comprehensive‐level health facilities serving the poorest areas in Honduras, Nicaragua, and Belize. Cases of severe pre‐eclampsia or eclampsia were randomly sampled and relevant quality of care data extracted from medical records at baseline (n=111) from January 1, 2011, to March 31, 2013, and at second‐phase follow‐up (n=249) from June 1, 2015, to September 30, 2017. The primary outcome was evidence of the delivery of multidimensional care for the management of pre‐eclampsia/eclampsia.

**Results:**

The care of 360 women with severe pre‐eclampsia or eclampsia was analyzed. Odds of multidimensional care for pre‐eclampsia management (*P*=0.271) increased (although not significantly) in the second‐phase follow‐up compared to baseline. Multidimensional care was significantly associated with training (*P*<0.001), basic‐level facilities (*P*<0.001), and higher in Honduras (*P*=0.001) and Belize (*P*=0.024) than the reference country of Nicaragua.

**Conclusion:**

Multidimensional care for pre‐eclampsia management increased across all facility types, countries, and severity of disease. The Salud Mesoamérica Initiative is a promising model for achieving such quality of care interventions in the era of universal health coverage.

## Abstract 2

Multidimensional care for pre‐eclampsia management increased across all facility types, participating countries, and severity of disease under the Salud Mesoamérica Initiative model in Central America.

## 1 INTRODUCTION

Pre‐eclampsia, defined as new‐onset hypertension after 20 weeks of pregnancy with signs of end‐organ dysfunction,^1^ is a major worldwide cause of maternal mortality, stillbirth, and neonatal morbidity such as asphyxia and prematurity.^2^ With advances in obstetric care in high‐income countries, such adverse outcomes from pre‐eclampsia are concentrated in low‐income countries.^3^, ^4^, ^5^ From 1990 to 2010, as stated in the Global Burden of Disease study, maternal hypertension became, or remained, the second leading cause of maternal death in Honduras, Nicaragua, and Belize.^6^

With the rise in the number of deliveries occurring at health facilities in low‐income countries, quality of care and multidimensional management for pre‐eclampsia are gaining attention.^3^, ^5^, ^7^, ^8^ Interventions are expanding beyond critical, single interventions such as magnesium sulfate administration.^3^, ^7^, ^8^ Given that timely identification of disease progression for pre‐eclampsia remains a challenge for providers,^1^, ^9^ other aspects of care for pre‐eclampsia that are vital for recognizing the severity of the disease, such as diagnostic examinations and earlier laboratory monitoring of end‐organ dysfunction, should be targeted, along with antihypertensive therapy.

Focusing on multidimensional care, the Salud Mesoamérica Initiative (SMI) is a public–private partnership that seeks to lower maternal and child mortality for the poorest population in Mesoamerica, 1.8 million women and children, by targeting specific indicators, or intervention goals.^10^, ^11^ Mesoamerica, a region that spans southern Mexico through Central America, shares similar health challenges in poor populations. For example, in 2011, the rate of receiving one skilled prenatal care visit in Honduras was 79% in poor areas compared to 97% by national estimates.^11^ SMI uses a “regional approach” to promote regional partnerships, inter‐country learning, and implement health‐related interventions.^12^ SMI covers a wide range of public health issues, such as preventive child health care, family planning, prenatal care, and emergency obstetric and newborn care (EONC).^13^

Through a stepwise approach, SMI puts into practice the WHO resolution of universal health coverage: improving quality of care, alongside access to care, for those at risk of financial hardship.^14^ The first phase of SMI, a year prior to 2013–2014 data collection, targeted availability of inputs and strengthening facility infrastructure. The second phase, a year prior to 2015–2017 data collection and presented in this study, aims to improve quality of care and coverage. Specifically, for pre‐eclampsia management, second‐phase indicators assessed a quality improvement bundle that focused on performing relevant examinations and laboratory testing for end‐organ monitoring, administering magnesium sulfate for seizure prevention and treatment, and appropriate use of antihypertensive drugs for blood pressure control.

The objective of the present study was to analyze multidimensional care practices for the management of pre‐eclampsia/eclampsia, before and after the implementation of SMI, in health facilities that serve the poorest communities of Central America. In doing so, we aimed to characterize the coverage and performance of crucial maternal care interventions in low‐resource settings.

## 2 MATERIALS AND METHODS

In the present evaluation study, information from the evaluation of the SMI was analyzed, with data collection periods occurring at the baseline (January 1, 2011, to March 31, 2013) and the second‐phase follow‐up (June 1, 2015, to September 30, 2017). In accordance with the EONC classification, facilities for this analysis were categorized into basic‐level facilities that offered fundamental care, such as providing essential medications and performing simple delivery‐related procedures, or comprehensive‐level facilities that offered higher‐level services, such as administering blood transfusions and performing surgery.^13^

Data were used from all 67 basic‐ and comprehensive‐level health facilities in SMI intervention areas serving the poorest areas. No sampling on the facility level was used due to the reduced number of facilities. These facilities were located in the municipalities of Bilwi, Jinotega, Las Minas, Matagalpa, and the North Atlantic Region (RAAN) in Nicaragua; the municipalities of Choluteca, Copan, Intibuca, La Paz, Lempira, Ocotepeque, and Olancho in Honduras; and the districts of Cayo, Corozal, and Orange Walk in Belize. In accordance with census data, the poorest areas were defined as consisting of the population in the lowest quintile of income. The initial SMI design stratified the poorest areas by intervention and control areas. However, due to limited real‐world resources, some countries chose to alter this design as the initiative evolved. For example, control data were not available at the baseline for Belize and at the second‐phase follow‐up for Nicaragua. Thus, the analysis was restricted to intervention areas. Exemption (non‐human‐subject research) and approval of this study were determined by the institutional review board from the University of Washington, along with partnering data‐collection agencies, and the ministry of health in each country. Written informed consent was acquired from health facility administrators prior to conducting health facility surveys. Medical records remained anonymized during data collection and during electronic uploading with DatStat Illume 6.1.16.17 (DatStat, Inc., Seattle, WA, USA).

For both data collection periods, a health facility survey was administered. The survey included an interview questionnaire on facility resources and personnel; an observation checklist of drug supplies and medical equipment; and a retrospective review of medical records on the health care services provided. Selection of medical records was based on international classification of diseases (ICD‐10) coding for severe pre‐eclampsia or eclampsia diagnosis. Records were sampled at random using a systematic sampling method to reach the required quota set for each facility. For the systematic sampling, records were chosen with a sampling interval equal to the size of the sampling fraction, beginning with a random starting point in time over the course of a 2‐year study period. Sampling quotas differed by round, facility type, and country. For example, more records were sampled in Nicaragua than in other countries to reflect a larger population size and magnitude of health problems. Data collection methodology for SMI is further detailed in earlier analyses.^11^, ^15^, ^16^, ^17^

The second phase of SMI concentrated on quality of care interventions, compared to the earlier first phase, not highlighted in this study, which invested in facility input interventions. Examples of second‐phase interventions included ensuring health staff training on emergency obstetric care within the last 3 years; improving protocols on emergency clinical scenarios; implementing quality improvement programs in hospitals; expanding referral networks; and establishing standardization of work processes.

**Figure 1:**
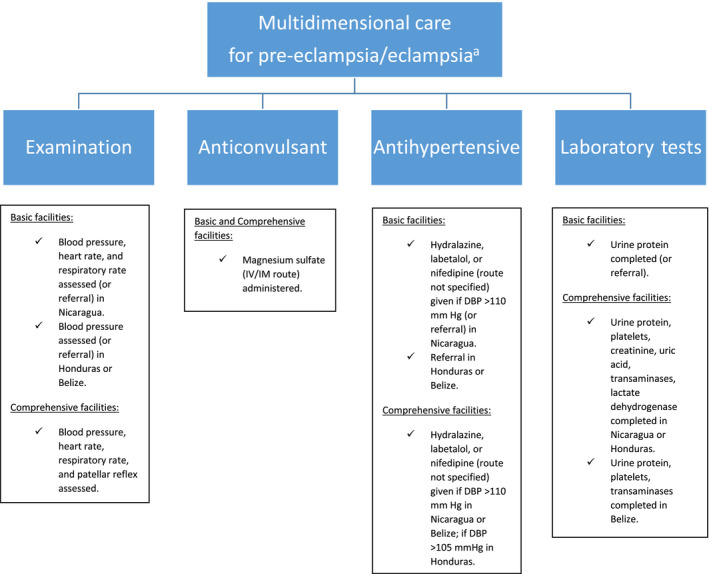
Definitions of Salud Mesoamérica Initiative (SMI) care practices for pre‐eclampsia/eclampsia. DBP, diastolic blood pressure; EONC, emergency obstetric and newborn care; IM, intramuscular; IV, intravenous. ^a^At first clinical check‐up in EONC health facility. Basic facilities = Health centers; Comprehensive facilities = Referral hospitals.

Multidimensional care package was implemented at first clinical checkup for those admitted to a health facility for pre‐eclampsia/eclampsia, consisting of four components: (1) examination performed for initial assessment of patient; (2) magnesium sulfate administered for seizure prevention or treatment; (3) antihypertensive therapy for blood pressure control; and (4) relevant laboratory tests completed for end‐organ monitoring. Multidimensional care components were based on the SMI indicators agreed upon by the ministries of health, reflecting national norms and international guidelines. Multidimensional care package component definitions by facility type and country, including indications for referral, are summarized in Figure 1.

Descriptive and multivariable logistic regression analysis was used. Covariates included in the analysis were timing of data collection (second‐phase follow‐up versus baseline); relevant training (EONC training within last 3 years versus none); EONC facility type (basic versus comprehensive); and severity of disease (eclampsia versus pre‐eclampsia). Regression models were adjusted for clustering of observations at the health facility level. *P*<0.05 was considered statistically significant. Stata 14.2 (StataCorp LP, College Station, TX, USA) was used for all analysis.

## 3 RESULTS

A total of 360 cases of severe pre‐eclampsia or eclampsia at 67 health facilities in Nicaragua, Honduras, and Belize were included in this analysis. Medical records, patient and facility characteristics are shown in Table 1. More pre‐eclampsia/eclampsia cases were located at comprehensive‐level facilities (205 [56.9%]). The most medical records were sampled from Nicaragua (180 [50.0%]), followed by Honduras (104 [28.9%]) and Belize (76 [21.1%]). Mean maternal age of patients was 24.0 years, ranging from 15 to 48 years. Of these women, 245 (86.3%) were married or in a social partnership, and 208 (76.2%) held less than a secondary education. The majority of women were diagnosed with pre‐eclampsia (314 [87.2%]), versus eclampsia (46 [12.8%]). Admission to a facility occurred less for those with preterm (<37 weeks) (145 [44.6%]) than those with full‐term (>37 weeks) gestational age (180 [55.4%]). If delivery was indicated, more resulted in cesarean delivery (196 [66.0%]) compared with vaginal delivery (101 [34.0%]). One maternal death was noted, and neonatal complications such as prematurity, low birth weight, or asphyxia occurred for 76 (21.1%) of all births. On the facility level, supply of magnesium sulfate, antihypertensive medications, and relevant laboratory equipment was available at 40 (97.6%), 41 (100.0%), 33 (100%) facilities respectively at the second‐phase follow‐up. Skilled personnel on staff was the highest for general physicians (40 [97.6%]), followed by nurses (38 [92.7%]), obstetricians (25 [61.0%]), and anesthesiologists (19 [46.3%]) at follow‐up. Relevant training within the last 3 years on pre‐eclampsia and eclampsia management, basic emergency obstetric care, or EONC, gained attendance at 40 (97.6%) facilities by second‐phase follow‐up.

**Table 1:** Medical records, patient and facility characteristics, by second‐phase follow‐up

	n (%)		
	Baseline 111 (30.8)	Second phase 249 (69.2)	Total 360 (100)
Medical record characteristics^a^			
EONC facility type			
Basic‐level	29 (26.1)	126 (50.6)	155 (43.1)
Comprehensive‐level	82 (73.9)	123 (49.4)	205 (56.9)
Country			
Nicaragua	53 (47.8)	127 (51.0)	180 (50.0)
Honduras	23 (20.7)	81 (32.5)	104 (28.9)
Belize	35 (31.5)	41 (16.5)	76 (21.1)
Severity of disease			
Pre‐eclampsia	97 (87.4)	217 (87.2)	314 (87.2)
Eclampsia	14 (12.6)	32 (12.9)	46 (12.8)
Patient characteristics^a^			
Maternal age^b^	23.6 ± 6.6	24.2 ± 7.1	24.0 ± 6.9
Marital status			
Single	19 (20.9)	20 (10.4)	39 (13.7)
Partnership	72 (79.1)	173 (89.6)	245 (86.3)
Education			
Less than secondary	67 (77.0)	141 (75.8)	208 (76.2)
Secondary or higher	20 (23.0)	45 (24.2)	65 (23.8)
Gestational age			
<37 wk	45 (46.4)	100 (43.9)	145 (44.6)
>37 wk	52 (53.6)	128 (56.1)	180 (55.4)
Delivery type			
Vaginal	28 (30.8)	73 (35.4)	101 (34.0)
Cesarean	63 (69.2)	133 (64.6)	196 (66.0)
Maternal outcome			
Alive	111 (100.0)	239 (99.6)	350 (99.7)
Dead	0 (0.0)	1 (0.4)	1 (0.3)
Neonatal outcome			
No complications recorded	81 (73.0)	203 (81.5)	284 (78.9)
Poor^c^	30 (27.0)	46 (18.5)	76 (21.1)

	n (%)		
	Baseline 26 (38.8)	Second phase 41 (61.2)	Total 67 (100)
Facility characteristics^d^			
Medication availability^e^			
Magnesium sulfate	24 (96.0)	40 (97.6)	64 (97.0)
Hydralazine, Nifedipine^f^	21 (84.0)	41 (100.0)	62 (93.9)
Laboratory equipment^g^	8 (88.9)	33 (100.0)	41 (97.6)
Personnel			
Nurse	23 (88.5)	38 (92.7)	61 (91.0)
General physician	26 (100.0)	40 (97.6)	66 (98.5)
Obstetrician	18 (69.2)	25 (61.0)	43 (64.2)
Anesthesiologist	11 (42.3)	19 (46.3)	30 (44.8)
Relevant training^h^	25 (96.2)	40 (97.6)	65 (97.0)

Abbreviation: EONC, emergency obstetric and newborn care.

a n may vary for each variable due to missingness.

b Mean ± SD; range 15–48 y.

c Asphyxia, low birth weight, prematurity, other complications.

d Percentages reflect the number of facilities within each round that had the input (e.g. medication availability, laboratory equipment, personnel, and relevant training). Not all inputs captured at every facility based on logic in the survey, therefore the denominator for each input does not always reflect total number of facilities visited at that round.

e Available on the day of the visit and no stock‐outs within last 3 mo.

f Labetalol data not available.

g Urine analysis and blood cell counter.

h Within last 3 y: includes management of pre‐eclampsia and eclampsia, basic emergency obstetric care, EONC training.

Overall, multidimensional care practices for pre‐eclampsia/eclampsia increased in SMI areas by 20.8 percentage points, from 22 (19.8%) at baseline to 101 (40.6%) cases by the second‐phase follow‐up. Table 2 shows intervention, country, facility, and patient determinants of multidimensional care for pre‐eclampsia/eclampsia. Odds of multidimensional care practices increased in magnitude at second‐phase follow‐up, but the increase was not significant after adjusting for covariates (odds ratio [OR] 1.73, 95% confidence interval [CI] 0.65–4.61). However, multidimensional care practices were found to be associated with training, which was one of the interventions focused upon during the second phase (OR 18.50, 95% CI 4.74–72.16).

**Table 2:** Factors associated with primary outcome

	Multidimensional care for pre‐eclampsia/eclampsia		
	Adjusted OR (n=360)	*P* value	95% CI
Second‐phase vs baseline	1.73	0.271	0.65–4.61
Relevant training vs none	18.50	0.001	4.74–72.16
Country			
Nicaragua	Ref	Ref	Ref
Honduras	12.48	0.001	3.01–51.81
Belize	2.87	0.024	1.15–7.19
Basic‐level vs comprehensive‐level facility	17.45	0.001	5.37–56.73
Eclampsia vs pre‐eclampsia	1.64	0.184	0.79–3.42

Abbreviations: CI, confidence interval; OR, odds ratio.

**Figure 2:**
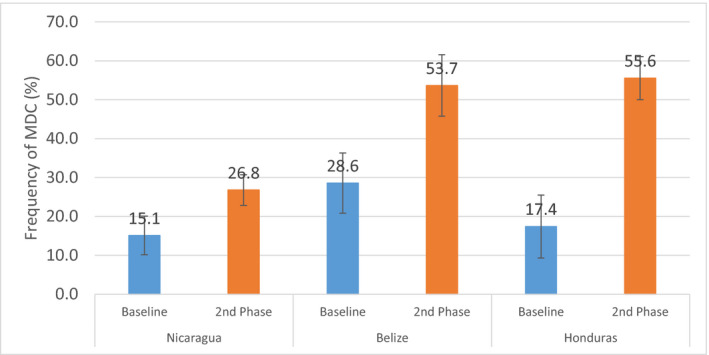
Multidimensional care for pre‐eclampsia/eclampsia, by country. MDC, multidimensional care.

At the country level, after adjustment for covariates, implementation of multidimensional care practices was significantly higher in Honduras (OR 12.48, 95% CI 3.01–51.81) and Belize (OR 2.87, 95% CI 1.15–7.19) than the reference country of Nicaragua (Table 2). While multidimensional care increased in all countries over time, Honduras and Belize reached coverage levels of 45 (55.6%) and 22 (53.7%) cases, respectively, compared with 34 (26.8%) cases in Nicaragua by the second‐phase follow‐up (Fig. 2). Additional country‐level results are noted in Figures S2–S5.

**Figure 3:**
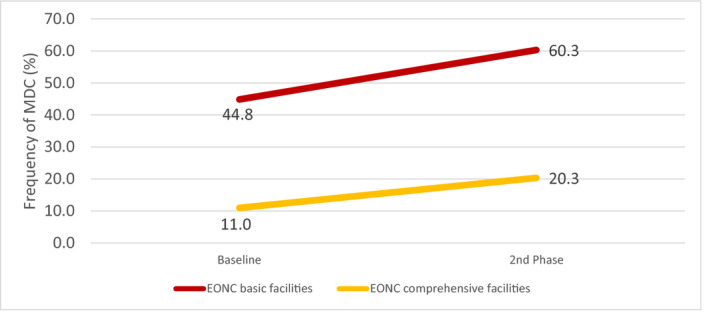
Multidimensional care for pre‐eclampsia/eclampsia, by EONC facility. EONC, emergency obstetric and newborn care; MDC, multidimensional care. Basic facilities = Health centers; Comprehensive facilities = Referral hospitals.

At the facility level, compliance of multidimensional care practices was significantly higher in basic‐level facilities (OR 17.45, 95% CI 5.37–56.73) as compared with comprehensive‐level facilities (Table 2). Both types of facilities increased multidimensional care over time. However, basic‐level facilities attained coverage levels of 76 (60.3%) cases, compared with 25 (20.3%) cases in comprehensive‐level facilities by the second‐phase follow‐up (Fig. 3). Additional facility‐level results are noted in Figures S6–S8.

**Figure 4:**
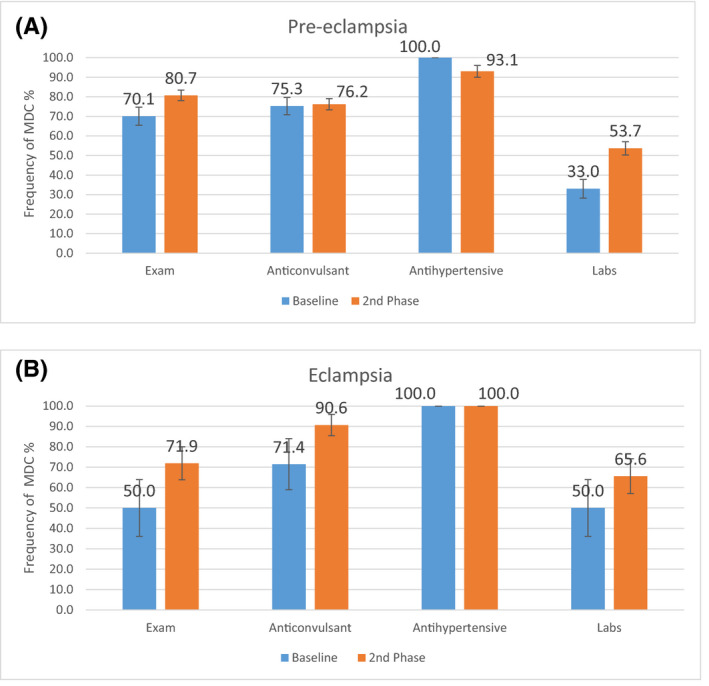
(A) Multidimensional care components, by disease severity (pre‐eclampsia). MDC, multidimensional care. Exam = (blood pressure, heart rate, respiratory rate, patellar reflex) or referral per country standards; Anticonvulsant = magnesium sulfate; Antihypertensive = (hydralazine, labetalol, or nifedipine) or referral per country standards; Labs = (urine protein, platelets, creatinine, uric acid, transaminases, lactate dehydrogenase) or referral per country standards. (B) Multidimensional care components, by disease severity (eclampsia). MDC, multidimensional care. Exam = (blood pressure, heart rate, respiratory rate, patellar reflex) or referral per country standards; Anticonvulsant = magnesium sulfate; Antihypertensive = (hydralazine, labetalol, or nifedipine) or referral per country standards; Labs = (urine protein, platelets, creatinine, uric acid, transaminases, lactate dehydrogenase) or referral per country standards.

At the patient level, multidimensional care increased regardless of disease severity, with compliance to multidimensional care for pre‐eclampsia and eclampsia groups reaching 85 (39.2%) and 16 (50.0%) cases, respectively, by the second‐phase follow‐up. Figures 4A,B present the components of multidimensional care by disease severity. Drugs (anticonvulsants and antihypertensives) increased or remained high for the sickest patients, at 29 (90.6%) and 15 (100.0%) cases, respectively, by second‐phase follow‐up. Examination and laboratory testing, which reflect recognition and monitoring of disease progression, gained the most progress from baseline, achieving levels ranging from 117 (53.7%) to 176 (80.7%) cases by the second‐phase follow‐up. Additional patient‐level results are noted in Figure S1.

## 4 DISCUSSION

To our knowledge, this is the first multinational study in Central America that assesses multidimensional care coverage of pre‐eclampsia/eclampsia management in health facilities. A few studies exist that examine quality‐improvement bundles for pre‐eclampsia/eclampsia patients in sub‐Saharan Africa,^3^, ^8^ and indirectly monitor multidimensional care for pre‐eclampsia within a single country in Central America^18^ On a broader level, literature also exists for low‐income countries on the practice of magnesium sulfate use alone^19^ and adverse outcomes of hypertensive disorders.^4^, ^5^, ^7^, ^20^ Thus, this study adds value in characterizing current coverage and performance of multidimensional care for pre‐eclampsia/eclampsia treatment specific to Central American health facilities.

The present study showed that, after SMI interventions, implementation of multidimensional care for pre‐eclampsia/eclampsia management in low‐resource facilities in Central America could be improved. Within the SMI model, multidimensional care not only increased overall by second‐phase follow‐up, but across all countries, all facility types, and the spectrum of disease severity (Table 2). In this region, maternal mortality rates as a result of pre‐eclampsia are lower than in parts of Asia and Africa,^6^ and therefore should expand beyond single interventions. This analysis highlights that the standard of care can reach the next level and incorporate a more comprehensive approach^3^, ^7^ for patients during hospitalization. In the era of universal health coverage put forth by WHO,^14^ SMI demonstrates the implementation of quality of care on a multinational level for pre‐eclampsia/eclampsia—an important part of the triad: quality of care, access to care, and receiving care without financial hardship—through a stepwise approach of strengthening health systems and exploring barriers to implementation.

In terms of a stepwise approach to health systems strengthening, SMI promotes multidimensional care for pre‐eclampsia/eclampsia management through three phases. In the first phase of interventions (prior to 2013–2014 data collection), SMI focused on system preparedness through improving supply chains, stock monitoring, and input availability. On the patient level, all drug, laboratory, and personnel‐related inputs from the first phase increased to or above 242 (97.2%) cases by second‐phase follow‐up (Fig. S1). These sustained effects from the first phase enabled prioritization of service coverage and quality of care in the second phase (prior to 2015–2017 data collection). Second‐phase interventions included ongoing training and emergency triage practices, optimizing referral systems, reviewing hospital protocol and processes, initiating quality‐improvement strategies, and use of diagnostics and monitoring, in addition to appropriate drug administration. Findings from the second phase, such as an increase in multidimensional care that had not reached significant levels by follow‐up (Table 2), as well as an improvement in end‐organ monitoring through examination and laboratory component increases by follow‐up (Figs 4A,B), suggest that more time might be needed to implement a multidimensional care package. Indeed, the goal of the final upcoming phase (projected 2020 data collection) would be to dedicate more time to reach significant levels of key interventions, while targeting identified barriers to implementation during this period.

Barriers that may be inhibiting the implementation of multidimensional care for pre‐eclampsia/eclampsia in SMI areas were evaluated. Nicaragua did not improve multidimensional care coverage at the same rate as Honduras and Belize for several reasons: more progress was needed with magnesium sulfate drug stock and laboratory equipment from baseline to second‐phase follow‐up, with an associated lower performance of anticonvulsant and laboratory testing components of the multidimensional care package; increased referrals by the second‐phase follow‐up; and facility expansions during the intervention period (Figs S2–S5).^21^ On the facility level, comprehensive facilities faced similar challenges compared to basic facilities: more progress was required for laboratory and relevant training inputs from baseline to the second‐phase follow‐up, with resultant lower completion of laboratory testing and clinical exam components of the multidimensional care package, respectively (Figs S6–S8). Last, despite same first‐phase inputs and training on the facility level, drugs were prioritized in eclampsia patients (Fig. 4B), rather than being equally administered across all patients.

The present study did have some limitations. First, while the initial SMI design planned for intervention and control groups, some ministries of health—due to limited real‐world resources—chose to focus country investment in intervention areas only. Consequently, there is no comparison group for all the countries in the study. It was decided to restrict the analysis to a pre–post design in all countries, without control groups, to provide a homogenous design. It is possible that changes in intervention areas may have been related to overall changes in the countries, and not exclusive to the effect of the intervention. However, SMI is a substantial (US $80 million) undertaking,^17^ with no other major maternal health intervention of a similar magnitude occurring in the region to our knowledge. In addition, we adjusted the analysis for clustering at the health facility level. To control for the effect of other characteristics of the women or health facility, we adjusted the main model by other covariates such as relevant training in the facility, level of the facility, and severity of disease. Second, multidimensional care component definitions varied among countries and by facility types (Fig. 1). However, these definitions were agreed upon by the ministries of health and reflect national norms. Slower rates of multidimensional care progress in Nicaragua and comprehensive facilities are accounted for in this section. Inclusion of referrals in the component definitions also added to this issue. Third, while most domains of quality of care, such as safe, effective, timely, and equitable care, were incorporated in this study via guideline adherence and medical record review, patient‐centered care was not assessed. The upcoming phase of SMI would benefit from a complementary client satisfaction survey to evaluate this valuable aspect of care.

In summary, SMI is a promising model to achieve improvements in multidimensional care for pre‐eclampsia/eclampsia management. SMI shows a positive trajectory for the implementation of multidimensional care of women with pre‐eclampsia/eclampsia on all levels and cumulative effects during the last two phases through health systems strengthening mechanisms. Based on second‐phase findings, it is necessary to maintain training and other quality improvement interventions, and to promote examination and laboratory components of a multidimensional care package as equally essential as drug administration for timely and comprehensive care. The present study provides valuable information on the current coverage and performance of crucial maternal care interventions in Central America, as well as offering next steps in attaining the goals of better recognition of disease progression and end‐organ dysfunction of pre‐eclampsia that are critical to reducing poor maternal and neonatal outcomes. Raising the standard of care for pre‐eclampsia management is feasible in low‐resource settings and should be promoted.

## AUTHOR CONTRIBUTIONS

AMK, DRZ, AHM, and BH contributed to the conception of the study. AMK contributed to theinterpretation and analysis of data, and writing the manuscript. CKJ, MGT, and RMC contributed to data collection and analysis. DRZ, AHM, and BH contributed to collection and interpretation of data. All authors contributed to revising the manuscript, and all authors approved the final manuscript.

## CONFLICTS OF INTEREST

The authors have no conflicts of interest.

## Supporting information


**Figure S1.** Facility inputs, by second‐phase follow‐up.
**Figure S2.** Magnesium sulfate stock input, by country.
**Figure S3.** Laboratory equipment stock input, by country.
**Figure S4.** Multidimensional Care (MDC) components, Nicaragua.
**Figure S5.** Referrals to higher‐level facilities, by country.
**Figure S6.** Laboratory equipment input, by facility type.
**Figure S7.** Training input, by facility type.
**Figure S8.** Multidimensional Care (MDC) components, in EONC comprehensive facilities.
