# Supplementary material for: Assessing multidimensional care coverage for pre‐eclampsia in the era of universal health coverage: A pre–post evaluation of the Salud Mesoamérica Initiative
Source: Int J Gynaecol Obstet. 2020 Mar 25;149(3):318–25. doi: 10.1002/ijgo.13131 (PMC7318157; doi:10.1002/ijgo.13131)
Supplement: Supplementary file 1 — Figure S1. Facility inputs, by second‐phase follow‐up. Figure S2. Magnesium sulfate stock input, by country. Figure S3. Laboratory equipment stock input, by country. Figure S4. Multidimensional Care (MDC) components, Nicaragua. Figure S5. Referrals to higher‐level facilities, by country. Figure S6. Laboratory equipment input, by facility type. Figure S7. Training input, by facility type. Figure S8. Multidimensional Care (MDC) components, in EONC comprehensive facilities. [file IJGO-149-318-s001.docx]

# Figure S1: Facility inputs, by second-phase follow-up

# Figure S2: Magnesium sulfate stock input, by country

# Figure S3: Laboratory equipment stock input, by country

# Figure S4: Multidimensional Care (MDC) components, Nicaragua

# Figure S5: Referrals to higher-level facilities, by country

# Figure S6: Laboratory equipment input, by facility type

# Figure S7: Training input, by facility type

# Figure S8: Multidimensional Care (MDC) components, in EONC comprehensive facilities
